# Withdrawal: Male‐to‐female and female‐to‐male transsexual siblings with pedophilic father: A case report

**DOI:** 10.1002/ccr3.9002

**Published:** 2024-05-24

## Abstract

Sheikhmoonesi, F. (2024) “Male‐to‐female and female‐to‐male transsexual siblings with pedophilic father: A case report” *Clinical Case Reports*, 12: e8580. https://doi.org/10.1002/ccr3.8580

The above article, published online on March 5, 2024 in Wiley Online Library (http://wileyonlinelibrary.com), has been withdrawn by agreement between the Journal's Editor‐in‐Chief, Dr Charles Young, and John Wiley & Sons, Ltd. The article came under investigation following reports by multiple third parties who expressed concerns about the patient consent described, as well as concerns about the methodology and the inclusion of potentially stigmatizing content. Significantly, the author included potentially identifiable information about the patients and family which was not fully appreciated during peer review. Upon request, the author provided copies of consent forms for two of the individuals described. An investigation by the publisher found that the consent forms did not conform to the Journal's nor Wiley's guidelines. Additionally, the author did not provide a consent form for one research subject whose private details were disclosed. In summary, through an investigation by the publisher, it was determined that patient consent had not been properly obtained and that anonymity had not been properly maintained in the published article, which would risk the identification of the patients and necessitated withdrawal. The author disagrees with the withdrawal.

